# Cardiovascular Risk and Disease Among Masters Endurance Athletes: Insights from the Boston MASTER (Masters Athletes Survey To Evaluate Risk) Initiative

**DOI:** 10.1186/s40798-016-0053-0

**Published:** 2016-08-09

**Authors:** Kayle Shapero, James Deluca, Miranda Contursi, Meagan Wasfy, Rory B. Weiner, Gregory D. Lewis, Adolph Hutter, Aaron L. Baggish

**Affiliations:** 1 Tufts University School of Medicine, Boston, MA USA; 2 Cardiovascular Performance Program, Massachusetts General Hospital, 55 Fruit Street, Yawkey Suite 5B, Boston, MA 02114 USA

**Keywords:** Exercise, Masters athlete, Sports cardiology, Cardiovascular risk factors

## Abstract

**Background:**

Masters athletes (MAs), people over the age of 35 that participate in competitive sports, are a rapidly growing population that may be uniquely at risk for cardiovascular (CV) disease. The objective of this study was to develop a comprehensive clinical CV profile of MA.

**Methods:**

An electronic Internet-based survey (survey response rate = 66 %) was used to characterize a community cohort of MAs residing in Eastern Massachusetts, USA. Clinical and lifestyle factors associated with prevalent CV disease were determined using logistic regression.

**Results:**

Among 591 MAs (66 % men, age = 50 ± 9 years) with 21.3 ± 5.5 years of competitive endurance sport exposure, at least one CV risk factor was present in 64 % including the following: family history of premature atherosclerosis (32 %), prior/current tobacco exposure (23 %), hypertension (12.0 %), and dyslipidemia (7.4 %). There was a 9 % (54/591) prevalence of established CV disease which was accounted for largely by atrial fibrillation (AF) and coronary atherosclerosis (CAD). Prevalent AF was associated with years of exercise exposure [adjusted odds ratio, OR (95 % confidence intervals); OR = 1.10 (1.06, 1.21)] and hypertension [OR = 1.05 (1.01, 1.10)] while CAD was associated with dyslipidemia [OR = 9.09 (2.40, 34.39)] and tobacco use [OR = 1.78 (1.34, 3.10)] but was independent of exercise exposure.

**Conclusions:**

Among MAs, AF is associated with prior exercise exposure whereas CAD is associated with typical risk factors including dyslipidemia and prior tobacco use. These findings suggest that there are numerous opportunities to improve disease prevention and clinical care in this population.

## Key Points

1. Approximately two thirds of community-based masters athletes have at least one established traditional cardiovascular risk factor with a family history of premature atherosclerotic disease and prior tobacco exposure identified as the most common issues.
2. Among community-based masters athletes, the estimated prevalence of established cardiovascular disease is a proximally 10 % and is accounted for almost exclusively by atrial fibrillation and atherosclerotic coronary artery disease.
3. Approximately one in seven masters athletes experience dissatisfaction with the healthcare system due in part to care providers’ tendency to dismiss complaints due to their status as athletes.

## Background

While habitual exercise reduces the risk of cardiovascular (CV) disease, it does not confer complete immunity [1, 2]. Masters athletes (MAs), men and women over the age of 35 who participate in organized competitive sport, are a rapidly growing segment of the population that are increasingly common in clinical practice [3]. Atherosclerotic coronary artery disease (CAD) is the most common cause of sudden death among MAs [4], and recent data suggest that MAs may be at higher risk for CAD than their otherwise similar sedentary counterparts [5, 6]. In addition, emerging data demonstrate associations between other forms of CV disease including atrial fibrillation (AF) and nonischemic myocardial fibrosis and exercise exposure in excess of low to moderate levels [7, 8]. However, lifestyle characteristics, CV risk factors, and their relationships with established CV disease among MAs remain incompletely understood.

To address this knowledge gap, we undertook the present study. Specifically, we utilized an Internet-based survey tool to collect demographic, lifestyle, and medical information from an unselected, community-based population of MAs. The primary goal of this initiative was to develop a comprehensive clinical profile of the contemporary MA with an emphasis on the relationship between CV risk factors and established disease.

## Methods

### Survey Design

The Boston Masters Athletes Survey To Evaluate Risk (MASTER) study utilized a web-based survey tool to collect data across complementary areas of interest in an effort to characterize a representative sample of MAs. The survey was comprised of 57 questions pertaining to demographics (*n* = 8), lifetime exercise exposure and dietary habits (*n* = 14), traditional CV risk factors (*n* = 11), personal health history (*n* = 12), and healthcare utilization and satisfaction (*n* = 12). The content, wording, and ordering of each survey question were designed to address issues of relevance as determined by a comprehensive literature search using the following keyword search terms: “masters athlete,” “older athlete,” “risk factors,” “cardiovascular risk factors,” and “cardiovascular disease,” and by aggregate clinical experience of the investigative team. Contents of the final survey were formatted for use in LimeSurvey (http://www.limesurvey.org, copyright © 2005), an open-source, fully customizable software package designed for research, clinical, academic, and operational data collection. Data defining survey content and participant responses were stored on Secure Sockets Layer (SSL)-encrypted servers maintained by Partners Healthcare’s Discovery Informatics Platform for Research (Newton, MA). All aspects of the survey, recruitment protocol, and analytic plan were approved by the Partner’s Human Research Committee. The study complied with the Declaration of Helsinki, and informed consent was obtained from each subject prior to beginning the survey.

Standardized definitions of CV risk factors were stipulated in the survey as follows. Hypertension was defined as the documentation of either a systolic blood pressure ≥140 mmHg or a diastolic blood ≥90 mmHg by a clinician on at least two occasions or ongoing use of one or more antihypertensive medications [9]. Dyslipidemia was defined as the laboratory-based documentation of a low-density lipoprotein level ≥160 on at least two occasions or ongoing use of one or more antihypertensive medications [10]. Prior tobacco use was defined as the previous daily use of a smokable or chewable tobacco product for >2 years. A significant family history of atherosclerosis was defined as the development of atherosclerotic CAD or death from CV disease in a first-degree relative prior to age 55 (males) or 65 (females). Similarly, established CV diseases were defined in the following standardized fashion. Atherosclerosis was defined by prior acute coronary syndrome, stroke, stable or unstable angina, or coronary/peripheral arterial revascularization. Arrhythmia was defined by the presence of one of the following: (1) second-degree, type 2, or complete heart block, (2) chronic persistent or paroxysmal AF or atrial flutter, or (3) an alternative sustained supraventricular or ventricular tachycardia as documented by 12-lead ECG, ambulatory rhythm monitoring, or hospital-based telemetry on at least one occasion. Valvular disease was defined as the prior echocardiographic documentation of stenosis or regurgitation of moderate or greater severity or a prior valve repair/replacement procedure.

### Survey Sample

Publically available, web-based search engines and the authors’ a priori knowledge of local organizations were used to identify MA clubs in the Greater Boston, MA, USA, area. We defined MA clubs as any publically advertised organization designed to facilitate sport-specific exercise training for older athletes with emphases on individual performance and/or team competition. Our geographic catchment area was limited both to the state of Massachusetts and to an 80-mile radius from central Boston. Following these criteria, we identified 28 eligible groups (Fig. 1). The chief administrative officer from each club was personally contacted via an introductory email that was used to delineate the goals of the survey and to determine general club interest in participation. Follow-up telephone correspondences were arranged on an individual basis as requested by the club’s chief administrative officer. Nonresponding club leaders were contacted a second time by email and/or telephone and were encouraged to participate. Of the 28 eligible clubs, 18 representing the following specific athletic disciplines: running (*n* = 3), cycling (*n* = 4), swimming (*n* = 4), rowing (*n* = 2), and triathlon (*n* = 5), ultimately confirmed willingness to participate. The 10 clubs that declined to participate represented the following sporting disciplines: running (*n* = 4), cycling (*n* = 2), rowing (*n* = 2), and triathlon (*n* = 2), and were all contained within the geographic catchment displayed in Fig. 1. Within the clubs that agreed to participate, the survey tool was distributed to 739 potential participants, which served as the source pool from which the final study sample was derived.

**Fig. 1:**
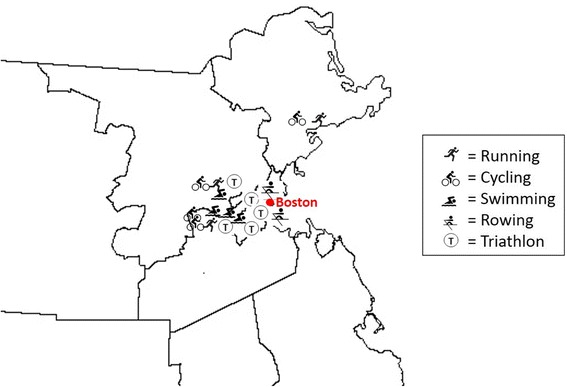
Geographical distribution of responding masters athlete organizations. Each of the 18 running, cycling, swimming, rowing, and triathlon teams who responded and agreed to disseminate the survey among their members are represented

### Survey Administration

Each participating club agreed to universally disseminate study-related information via member email distribution lists to official club members. Between July 2014 and October 2014, athletes belonging to one of the clubs described above received an introductory letter via email along with a secure link to the electronic survey. The survey was accessible for a 6-week time period. Participants were asked to complete the survey in its entirety but were permitted to do so in multiple sittings to facilitate confirmation of data in cases where medical record reviews or contact with personal physicians were required. Complete participant anonymity was maintained as per the commercially available software that is designed to keep all identifying information including respondents IP addresses shielded from investigators. Submitted surveys that were less than 95 % complete and those that were filed by athletes under the age of 35 were excluded from the final database.

### Statistical Analysis

Data were manually extracted from the survey tool into Excel spreadsheets. Continuous variables are presented as means ± standard deviations and categorical variables as fractions. Gender-based comparisons were made using two-tailed unpaired *t* tests, chi-squared tests, or Fischer’s exact test as appropriate for data distribution. Univariate and multivariate logistic regression were used to determine factors associated with prevalent atherosclerotic CAD and cardiac arrhythmia. Factors with a univariate *p* value of less than 0.10 were tested in forward step-wise multivariate logistic regression analyses to identify independent predictors of these two forms of CV disease (Table 5). Reported odds ratios (ORs) reflect risk as dictated by the binary presence of selected dependent variables (i.e., established hypertension vs. no hypertension) or per year in the context of the dependent variable “years of exercise exposure.” Analyses were performed with the use of SPSS software (version 21, IBM). A *p* value of <0.05 was considered significant.

## Results

### Baseline Demographics

The survey tool was sent to 739 athletes with a final sample yield consisting of 591 participants (men, *n* = 391; 66 %). Attrition was accounted for by incomplete survey data (*n* = 32) and failure to reply to the invitation to participate (*n* = 116). Demographics for the total sample and as stratified by gender are shown in Table 1. Compared to female participants, men were significantly older (51 ± 9 vs. 48 ± 9 years, *p* < 0.001) and were more likely to be married or in a committed monogamous relationship (89 vs. 71 %, *p* < 0.001). Of the participants, 34 % (201/591) used ≥1 prescription medication on a daily basis. The most commonly used medications included lipid lowering agents (used by 8.9 % of men, 2.5 % of women; *p* = 0.003), antidepressants (used by 3.6 % of men, 12.5 % of women; *p* < 0.001), antihypertensives (used by 6.6 % of men, 5.0 % of women; *p* = 0.427), thyroid replacement therapy (used by 3.1 % of men, 11.0 % of women; *p* < 0.001), and anticoagulants including aspirin (used by 6.6 % of men, 2.0 % of women; *p* = 0.003).

**Table 1:** Baseline demographics

	Total	Men (*n* = 391)	Women (*n* = 200)	*p* value
Age, years	50 ± 9	51 ± 9	48 ± 9	0.001
Height, cm	174.8 ± 13.7	179.3 ± 11.2	165.9 ± 13.7	<0.001
Weight, kg	72.9 ± 13.0	78.6 ± 10.7	61.9 ± 9.8	<0.001
Body mass index, kg/m^2^	23.4 ± 3.6	22.4 ± 2.8	24.0 ± 3.8	<0.001
Marital status				
Married/committed relationship	490/591 (82.9)	349/391 (89.3)	141/200 (70.5)	<0.001
Single	52/591 (8.8)	24/391 (6.1)	28/200 (14.0)	0.001
Divorced	47/591 (8.0)	18/391 (4.6)	29/200 (14.5)	<0.001
Children	426/591 (72.1)	314/391 (80.3)	112/200 (56.0)	<0.001
Religious	200/591 (33.8)	136/391 (34.8)	64/200 (32.0)	0.498
Medication use				
Lipid lowering	40/591 (6.8)	35/391 (8.9)	5/200 (2.5)	0.003
Antidepressants	39/591 (6.6)	14/391 (3.6)	25/200 (12.5)	<0.001
Blood pressure medications	36/591 (6.1)	26/391 (6.6)	10/200 (5.0)	0.427
Thyroid replacement therapy	34/591 (5.8)	12/391 (3.1)	22/200 (11.0)	<0.001
Aspirin/anticoagulants	30/391 (5.1)	26/391 (6.6)	4/200 (2.0)	0.003
Pain medications	19/591 (3.2)	11/391 (2.8)	8/200 (4.0)	0.465
Asthma/allergy	19/591 (3.2)	10/391 (2.6)	9/200 (4.5)	0.223
Other^a^	18/591 (3.0)	11/391 (2.8)	7/200 (3.5)	0.622
OC/hormone replacement therapy	10/591 (1.7)	0/391 (0.0)	10/200 (5.0)	N/A
BPH medications/sildenafil	5/591 (0.8)	5/391 (1.3)	0/200 (0.0)	N/A
Arrhythmia drugs	3/591 (0.5)	2/391 (0.5)	1/200 (0.5)	1.000

Values are mean ± SD or *n* (%); *p* values reflect significance of difference between men and women

*BPH* benign prostatic hyperplasia, *OC* oral contraceptive, *N/A* not applicable

^a^Includes allopurinol, antiseizure, biologics, and immunosuppressants

### Exercise Exposure and Lifestyle Habits

Lifestyle habits and exercise exposure history are shown in Table 2. Participants reported 21.3 ± 12.9 years of prior participation in competitive endurance sports. Cycling was the most common primary sport among men while running was the most common primary sport among women. Participants reported 10 ± 6 h of weekly total exercise training of which approximately 60 % was dedicated to their primary sport. Active tobacco use was rare (men 2/391, <1 %; females 1/200, <1 %; *p* = 0.73), but 23 % were prior tobacco users. Compared to women, men drank more alcohol on a weekly basis and were more likely to have been told by a friend or a peer that their alcohol consumption was problematic. Finally, approximately one in six participants adhered to some form of macronutrient dietary restriction, and the majority (men = 68 %, women = 74 %; *p* = 0.152) reported routinely using diet to control weight.

**Table 2:** Lifestyle habits, exercise exposure, and dietary preferences

	Total	Men	Women	*p* value
Participation in endurance sports, years	21.3 ± 132.9	22.4 ± 13.3	19.2 ± 121.6	0.003
Participation in high school sport	441/591 (74.6)	306/391 (78.3)	135/200 (67.5)	0.004
Participation in college sport	282/591 (47.7)	204/391 (52.2)	78/200 (39.0)	0.002
Current primary sport				
Cycling	246/591 (41.6)	201/391 (51.4)	45/200 (22.5)	<0.001
Running	147/591 (24.9)	76/391 (19.4)	71/200 (35.5)	<0.001
Swimming	72/591 (12.2)	33/391 (8.4)	39/200 (19.5)	<0.001
Triathlon	54/591 (9.1)	33/391 (8.4)	21/200 (10.5)	0.411
Rowing	56/591 (9.5)	38/391 (9.7)	18/200 (9)	0.778
Other^a^	11/591 (1.9)	7/391 (1.8)	4/200 (2.0)	<0.001
Current total training, h/week	10.3 ± 5.5	10.1 ± 4.5	10.6 ± 7.1	0.298
Weekly training breakdown, avg. h/week				
Primary sport	6.5 ± 4.4	6.8 ± 4.0	6.1 ± 5.3	0.111
Alternative endurance activity	1.9 ± 2.6	1.6 ± 2.1	2.6 ± 3.4	<0.001
Strength training	0.6 ± 1.0	0.6 ± 0.9	0.8 ± 1.1	0.027
Other^a^	0.5 ± 1.3	0.4 ± 1.1	0.7 ± 1.6	0.029
Tobacco exposure				
Former smoker	134/591 (22.7)	87/391 (23.8)	47/200 (25.5)	0.731
Current smoker	3/591 (0.5)	2/391 (0.5)	1/200 (0.5)	1.000
Alcohol consumption				
Total number of drinks/week	4.7 ± 4.8	5.3 ± 5.2	3.7 ± 3.9	<0.001
Number of nights/week with ≥1 drink	2.7 ± 2.4	2.9 ± 2.4	2.4 ± 2.3	0.015
Personal/peer concern about over-drinking	94/591 (15.9)	72/391 (18.4)	22/200 (11.0)	0.020
Dietary habits				
Routinely use diet to control weight	412/591 (69.7)	265/391 (67.8)	147/200 (73.5)	0.152
Follow strict diet^b^	103/591 (17.4)	63/391 (16.1)	40/200 (20.0)	0.239

Values are mean ± SD or *n* (%); *p* values reflect significance of difference between men and women

^a^Includes boot camp, P90X, weight training, and yoga

^b^Includes gluten-free, lactose-free, and paleo diet, vegan, and vegetarian

### Healthcare Utilization and Satisfaction

Data describing healthcare utilization and satisfaction are shown in Table 3. Approximately three quarters of the sample reported attending visits to a primary care physician on an annual basis (men = 75 % vs. women = 79 %, *p* = 0.302), and approximately one third of the sample had received care from a CV specialist on at least one occasion (men = 38 % vs. women = 24 %, *p* < 0.001). Exposure to prior CV diagnostic testing is shown in Fig. 2. Among men, 63 % reported having undergone at least one prior CV test compared with a 45 % test exposure rate among women (*p* < 0.001). Twelve-lead electrocardiography was the most commonly utilized test followed in decreasing order by exercise stress testing, transthoracic echocardiography, and cardiac CT scanning. For each of these testing modalities, rates of prior testing exposure were higher for men than women.

**Table 3:** Healthcare utilization and satisfaction

	Total	Men	Women	*p* value
Regularly visit a PCP	452/591 (76.5)	294/391 (75.2)	158/200 (79.0)	0.302
≥1 visit to a cardiologist	195/591 (33.0)	148/391 (37.9)	47/200 (23.5)	<0.001
≥1 visit to a sports med specialist	353/591 (59.7)	230/391 (58.8)	123/200 (61.5)	0.530
≥1 visit to a nutritionist	113/591 (19.1)	57/391 (14.6)	56/200 (28.0)	<0.001
Dissatisfied with current healthcare	80/591 (13.5)	49/391 (12.5)	31/200 (15.5)	0.318
Provider type responsible for dissatisfaction				
Primary care provider	60/80 (75.0)	36/49 (73.5)	24/31 (77.4)	0.794
Subspecialist	9/80 (11.3)	5/49 (10.2)	4/31 (12.9)	0.729
Nonphysician provider	3/80 (3.8)	3/49 (6.1)	0/31 (0)	0.279
Other	8/80 (10.0)	5/49 (10.2)	3/31 (9.7)	1.000
Primary reason for dissatisfaction				
Concerns dismissed due athlete status	17/80 (21.3)	7/49 (14.3)	10/31 (3.2)	0.091
Not enough time/attention paid	8/80 (10.0)	8/49 (16.3)	0/31 (0)	N/A
Minimal knowledge of athlete issues	8/80 (10.0)	8/49 (16.3)	0/31 (0)	N/A
Cost	7/80 (8.8)	5/49 (10.2)	2/31 (6.5)	0.700
Access	7/80 (8.8)	4/49 (8.2)	3/31 (9.7)	1.000
Misdiagnosis/no resolution of problem	6/80 (7.5)	2/49 (4.1)	4/31 (12.9)	0.200
Prescribed med impaired performance	5/80 (6.3)	3/49 (6.1)	2/31 (6.5)	1.000
Counseled to reduce exercise	5/80 (6.3)	4/49 (8.2)	1/31 (3.2)	0.644
Counseled to terminate exercise	4/80 (5.0)	2/49 (4.1)	2/31 (6.5)	0.639
Not indicated	13/80 (16.3)	6/49 (12.2)	7/31 (22.6)	0.234

Values are *n* (%); *p* values reflect significance of difference between men and women

**Fig. 2:**
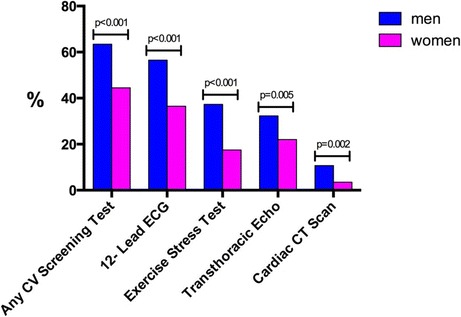
Prior exposure to cardiovascular screening/diagnostic tests. Among respondents, men underwent significantly more CV screening tests than women both in total and across the four different testing modalities. *CT* computed tomography, *ECG* electrocardiography

Indices reflecting satisfaction with the healthcare system are shown in Table 3. We observed an overall rate of healthcare system dissatisfaction of approximately 14 %. Among participants who reported dissatisfaction, the most common reasons for dissatisfaction included (1) presenting concerns dismissed due to status as an athlete (21 %), (2) inadequate time and/or attention paid during clinical visits (10 %), and (3) inadequate knowledge among care providers of issues specific to athletes (10 %).

### Cardiovascular Disease Risk Factors

The prevalence of CV disease risk factors is detailed in Table 4. A significant family history of atherosclerosis (32 % of respondents) and prior tobacco use (23 % of respondents) were the most commonly reported risk factors. Hypertension (men = 15 % vs. women = 6 %, *p* = 0.001) and dyslipidemia (men = 10 % vs. women = 3 %, *p* = 0.001) were more common among men than women. Notably, only 27 % of MAs were completely free of CV risk factors while roughly two thirds had one or more asymptomatic risk factors as shown in Fig. 3.

**Table 4:** Prevalence of traditional CV risk factors and established CV disease

	Total	Men	Women	*p* value
CV risk factors				
Family history of atherosclerosis	189/591 (32.0)	120/391 (30.7)	69/200 (34.5)	0.347
Former/current smoker	137/591 (23.2)	89/391 (22.7)	48/200 (24)	0.736
Hypertension	71/591 (12.0)	59/391 (15.1)	12/200 (6.0)	0.001
Dyslipidemia	44/591 (7.4)	38/391 (9.7)	6/200 (3.0)	0.003
Obesity	17/591 (2.9)	14/391 (3.6)	3/200 (1.5)	0.58
Diabetes mellitus	2/591 (0.3)	2/391 (0.5)	0/200 (0.0)	N/A
Aggregate CV disease	54/591 (9.1)	40/391 (10.7)	14/200 (8.0)	0.237
Arrhythmia	26/54 (48.1)	21/40 (52.5)	5/14 (35.7)	0.358
Atherosclerosis	16/54 (29.6)	13/40 (32.5)	3/14 (21.4)	0.516
Valvular	10/54 (18.5)	4/40 (10.0)	6/14 (42.9)	0.013
Other^a^	2/54 (3.7)	2/40 (5.0)	0/14 (0.0)	N/A

Values are *n* (%); *p* values reflect significance of difference between men and women

*CV* cardiovascular

^a^Includes one case of pericarditis and one case of atrial septal defect

**Fig. 3:**
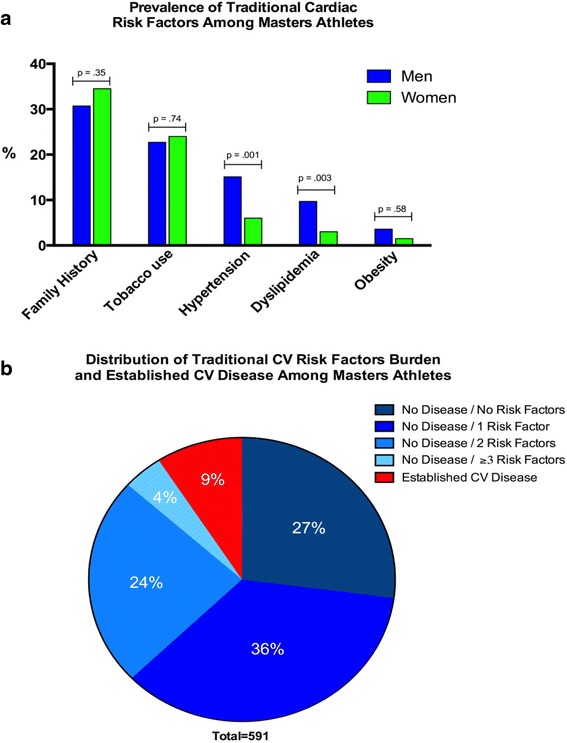
**a** Prevalence of traditional cardiovascular risk factors and **b** distribution of risk factor burden and disease prevalence. **a** Significant family history of atherosclerosis and prior/current tobacco exposure were the most common CV risk factors among male and female MAs. Men had significantly higher rates of hypertension and dyslipidemia, as compared to women. **b** Graphical representation of the survey population stratified by risk factor and disease burden. Of this population, 64 % reported at least one cardiovascular risk factor while the overall prevalence of established CV disease was 9 %. *CAD* coronary artery disease

### Established Cardiovascular Disease

The prevalence of established CV disease was 9 % (54/591) among this community-based cohort of MAs. Arrhythmia (26/591, 4.4 %), accounted for almost exclusively by AF (24/26 cases), and atherosclerosis (16/591, 2.7 %), accounted for exclusively by CAD, were the two most common conditions and were similarly prevalent among men and women. The results of logistic regression analyses used to identify factors associated with arrhythmia and CAD are shown in Table 5. In multivariate logistic regression models controlling for age, cumulative years of exercise exposure [OR = 1.10 (1.06, 1.21)] and hypertension [OR = 1.05 (1.01, 1.10)] were independently associated with the presence of AF, while in contrast, dyslipidemia [OR = 9.09 (2.40, 34.39)] and prior tobacco use [OR = 1.78 (1.34, 3.10)] were independently associated with prevalent CAD.

**Table 5:** Univariate and multivariate logistic regression analyses demonstrating factors associated with established arrhythmia and coronary artery disease

	Arrhythmia (*n* = 26)		Coronary artery disease (*n* = 16)	
	Univariate analysis	Multivariate analysis	Univariate analysis	Multivariate analysis
	OR (95 % CI)	OR (95 % CI)	OR (95 % CI)	OR (95 % CI)
Age	1.07 (1.02, 1.12)	1.06 (1.00, 1.11)	1.08 (1.02, 1.15)	1.06 (1.01, 1.14)
Male gender	2.07 (0.68, 6.35)	–	2.46 (0.53, 11.5)	–
Years exercise exposure	1.14 (1.08, 1.27)	1.10 (1.06, 1.21)	1.01 (0.97, 1.06)	–
Exercise (h/week)	1.01 (0.94, 1.08)	–	0.99 (0.87, 1.12)	–
Alcohol (drinks/week)	1.26 (1.09, 1.93)	–	0.94 (0.80, 1.09)	–
Prior/active tobacco use	1.08 (0.97, 1.18)	–	3.56 (1.35, 5.78)	1.78 (1.34, 3.10)
Family history	0.79 (0.28, 2.23)	–	2.75 (0.83, 9.16)	–
Hypertension	1.07 (1.02, 1.12)	1.05 (1.01, 1.10)	1.08 (1.02, 1.15)	–
Dyslipidemia	1.95 (0.54, 6.99)	–	13.92 (4.04, 47.91)	9.09 (2.40, 34.39)

## Discussion and Conclusions

The Boston MASTER study was conducted to develop a comprehensive CV profile of the contemporary MA. Key findings from this study, a characterization of men and women with more than 20 years of competitive endurance sport exposure who at the time of this study were completing approximately four times the amount of physical exercise recommended for health optimization [11], can be summarized as follows. First, 64 % of this population had at least one established CV risk factor with a family history of premature atherosclerotic disease and prior tobacco exposure identified as the most common issues. Second, there was a 9 % prevalence of established CV disease that was accounted for almost exclusively by AF and CAD. Third, roughly one in seven MAs reported overall dissatisfaction with the healthcare system due in part to care providers’ tendency to dismiss complaints due to their status as athletes. Finally, our data shed important insights into CV disease characteristics among MAs. Specifically, prevalence of AF appears to be associated with years of cumulative exercise exposure and concomitant hypertension, while in contrast, CAD appears to be associated with traditional atherosclerotic risk factors (i.e., hypertension, dyslipidemia) and negligibly associated with any element of prior exercise exposure.

Routine low to moderate intensity physical exercise leads to favorable changes in CV risk factors [12–14] and is recommended for both the primary and secondary prevention of CV disease [11]. However, the dose response relationship between exercise and CV outcomes remains incompletely understood, and there is mounting evidence that people who engage in high levels of exercise may be surprisingly susceptible to specific forms of heart disease including AF [7], coronary atherosclerosis [6], and myocardial fibrosis [15]. In addition, two recent observational population-based studies suggest that the mortality benefits afforded by physical activity may dissipate among people who engage in the highest levels of exercise [16, 17]. Definitive explanations for these intriguing observations remain speculative, in part because studies aimed at examining the people who engage in the highest levels of exercise have been lacking. Findings from this study present a comprehensive social and CV medical profile for this segment of the population and shed novel insights into why high-level exercisers may be susceptible to common forms of heart disease.

Our data support the notion that AF is the most common acquired CV condition among MAs and complement recent work demonstrating an association between duration of participation in competitive athletic and susceptibility to arrhythmia [7, 18]. In addition, we now demonstrate that concomitant hypertension is independently associated with prevalent AF. Future prospective interventional studies will be required to determine whether aggressive blood pressure control will lead to a reduced burden of AF among chronic high-level exercisers. Unlike AF, we detected no associations between atherosclerotic CAD and exercise exposure. This finding was somewhat surprising following recent reports of increased CAD among seasoned marathon runners [5, 6]. While we cannot exclude the possibility that high-level exercise plays a role in the progression of atherosclerosis, our data suggest that the presence and perhaps undertreatment of traditional atherosclerotic risk factors are a far more powerful determinant of CAD risk. This finding helps to explain why CAD occurs among seasoned exercisers and simultaneously provides opportunities to improve primary prevention efforts in clinical practice including the targeted treatment of modifiable atherosclerotic risk factors.

Existing data examining CV risk factor prevalence among competitive MAs are sparse. De Matos et al. reported age-dependent increases in the rates of abnormal lipid profiles, body mass index, and fasting glucose among 247 Brazilian MAs [19]. Risk factor prevalence data from the present study builds on this prior work in several important ways. The nontrivial rates of hypertension (12.4 %), dyslipidemia (7.4 %), and obesity (2.9 %) observed among this current cohort of MAs are substantially lower than those recently reported in the general population [20]. Thus, long-term dedication to high levels of exercise may substantially reduce the burden of these disease determinants but does not appear to eliminate them completely. In contrast, the rate of tobacco exposure observed in our cohort of MAs is similar to that reported in the general population. Our finding that prior tobacco exposure was an independent risk factor for CAD indicates that it is a significant source of morbidity among MAs. While speculative, it is likely that some older athletes adopt a high-level exercise lifestyle following years of previous risk exposure in an attempt to offset previous high-risk lifestyle choices. Clinical characterization of overall risk and future studies geared toward delineating disease pathogenesis in this population should take measures to address this issue.

To our knowledge, healthcare utilization and satisfaction have not been previously characterized among MAs. It is noteworthy that approximately one in four of our study participants did not receive routine primary care. This finding, coupled with the observed prevalence of both risk factors and disease, suggests a need for more effective public health outreach campaigns that should be designed to complement current screening recommendations for this specific population [3, 21]. Among those athletes who were engaged in the healthcare system, one third had received care from a CV specialist and 63 % had been subjected to one or more forms of CV diagnostic testing. These findings highlight the need for CV clinicians to be familiar with the unique characteristics of this population and emphasize the importance of developing MA-specific algorithms for the implementation and interpretation of CV testing. Optimal care of aging athletes may best be accomplished through a partnership between sports medicine physicians and CV specialists with expertise in the care of athletes. Finally, we observed a rate of overall healthcare dissatisfaction of 14 % with several underlying explanations that are unique to this population. Specifically, participants reported dissatisfaction due to (1) having their concerns dismissed due to their status as ostensibly healthy athletes and (2) inadequate knowledge among care providers about issues specific to athletes. These reasons for dissatisfaction indicate a previously unrecognized form of patient-provider mismatch that may be addressed by refinements in clinical training and further development of sports cardiology as an established area of expertise.

We acknowledge several limitations of this study. Data acquisition was performed using an Internet-based survey, and therefore, several potential sources of error must be addressed [22]. We sought to minimize the impact of nonresponse error by excluding all data derived from surveys that were less than 95 % complete. Similarly, we sought to minimize the likelihood of inaccurate response data by utilizing concise and clearly worded survey questions coupled with very specific trait definitions of cardiac risk factors and disease to reduce the impact of measurement error. As we studied MAs from a relatively confined geographic area, we acknowledge the possibility of coverage error that may limit the generalizability of our results to MAs in other locations. Similarly, we are unable to exclude some element of sampling error as our data reflect voluntary survey participation and it is possible that respondents on either end of the health spectrum were more or less likely to participate. However, we attempted to reduce sampling bias by advertising this survey as a general exercise and lifestyle study, thereby minimizing selection as a function of health status, and made our survey available to a large unselected group of potential participants. Finally, given the observational and cross-sectional nature of our data and the relatively low incidence of established disease, we are unable to determine the clinical significance and modifiability of the reported ORs.

People including MAs who engage in high levels of routine physical exercise are not completely protected from CV disease. AF and CAD have emerged as important sources of morbidity and mortality in this segment of the population and appear to be associated with distinctly different determinants of disease pathogenesis. Widespread recognition of disease-specific risk profiles coupled with targeted therapeutic efforts among MAs may hold value for preventing the development of CV disease in this population.
